# Screening of antibiofilm and anti-quorum sensing activty of Actinomycetes isolates extracts against aquaculture pathogenic bacteria

**DOI:** 10.1186/s12866-020-02022-z

**Published:** 2020-11-12

**Authors:** Gloria Raissa, Diana Elizabeth Waturangi, Dinamella Wahjuningrum

**Affiliations:** 1 Master of Biotechnology Program, Faculty of Biotechnology, Atma Jaya Catholic University of Indonesia, Jalan Jenderal Sudirman, Jakarta, 12930 Indonesia; 2 Department of Aquaculture, Faculty of Fisheries and Marine Science, Bogor Agricultural University, Jalan Raya Dramaga, Bogor, 16680 Indonesia

**Keywords:** *Actinomyces*, Antibiofilm, Aquatic pathogen, Biofilm, Quorum sensing

## Abstract

**Background:**

Indonesia is the third largest producer of fish and other aquaculture products in the world, making this industry a major contributor in the economy of Indonesia. However, this industry continually overcome challenges, one of them are bacterial outbreaks. In addition, the emergence of these bacterial outbreaks were worsen due to the biofilm produced by many significant pathogenic bacteria and the impact of increased antibiotic resistance. These issues have become a global concern, because antibiotics are currently one of the main treatments available to overcome this problems. Therefore, studies aimed at finding and characterizing bioactive compounds to combat these issues. In this study actinomycetes isolates were screened and characterized for their bioactive compounds produced which have inhibitory and destructive activity and also QS inhibitors against biofilm structure of aquatic pathogenic bacteria, such as *Vibrio harveyi*, *A. hydrophila*, and *S. agalactiae*.

**Result:**

Extracts (20 mg/mL) produced by sixteen Actinomycetes isolates showed anti-quorum sensing activity towards reporter stain *Chromobacterium violaceum* wild-type. Most of these extracts showed better inhibitory activity on all of the pathogenic bacteria biofilm structure tested than the destructive activity on the preformed of those biofilm structure. Subsequently, we also performed characterization of bioactive compound and found that in this study, polysaccharide is the most common antibiofilm agents, which were responsible to their antibiofilm activity. Finally, we found that the value of LC_50_ of all extracts tested were more than 1 mg/mL, thereby all of extracts tested did not show cyto-toxic effect against *Artemia salina*.

**Conclusion:**

All of the extracts of Actinomycetes isolates showed promising inhibitory activity towards biofilm structure of pathogenic bacteria tested. So far, all of the extracts are potential to be QS inhibitors and antibiofilm agents of all pathogenic bacteria tested.

## Background

The aquaculture industry is one of the main producers in the food sector globally by providing high-protein food source for world population. In fact, capita food fish consumption increased by 1.5% per year from 9.0 kg in 1961 to 20.5 kg in 2018. Indonesia is the third largest producer of fish aquaculture products in the world, which it has been estimated that Indonesia had produced 5.4 million tons of fishes, 3.5 million tons of aquaculture products including aquatic plants, and 0.9 million tons of finfish until 2018. In addition to that, it is known that Indonesia is the second largest producer of crustaceans producing 0.9 million tons of them [1]. However this industry is continually overcoming the same challenges, one of them are bacterial outbreaks, causing crop failures in the aquaculture industry and income loss. This emergence of these bacteria outbreaks also cause problems especially due to the biofilm produced by several pathogenic bacteria and its impact of increased antibiotic resistance [2].

Biofilms are bacterial multispecies communities, which attach to a surface and are embed by extracellular polymeric substances (EPS). These communities are formed as a bacterial response in the face of hostile environments, such as nutritional deficiencies, desiccation, and high frequency antibiotic and disinfectant exposure. The formation of biofilm structure is known to have implications for the increase in bacterial resistance to immune system of the host and antimicrobial agents, one of which is antibiotics, up to 1000 times the normal dose [3]. This is due to the nature of the sessile cells (cells which live within the biofilm structure) and biofilm structure. For example, the low nutrient state in the biofilm structure could result in cell dormancy, where the rate of cell metabolic is very low, causing cells to become insensitive to particular type of antibiotics, such as β-lactam [4]. In addition, close cell contact within biofilm structure is known to increase the efficiency of horizontal gene transfer, resulting in increased the spread of genes associated with resistance [5].

In the aquaculture system, biofilm structures were also found. It is known that there are many fish pathogenic bacteria which also have the ability to form these structures, such as *Vibrio harveyi* and *Aeromonas hydrophila*. It has become a concern, because biofilm structures can act as reservoirs of those bacteria populations and this structure makes the bacteria become more resistant to antibiotics [6]. This also has become major concern, considering antibiotics are major front liner treatment in the face bacterial outbreak. In addition, the prolonged use of antibiotics in aquaculture could increase the spread of resistance genes even to humans. Due to the nature of antibiotics being relatively stable and non-biodegradable, thereby the residual of these compounds could remain in the aquaculture product for human consumption [7].

The process of biofilm formation is regulated by a cell to cell communication, called quorum sensing (QS) system. This system is also involved in various regulations of many gene expressions, such as bioluminescence, secretion of virulence factors, and formation of biofilm structures [8], making this system a promising target in dealing with infections and bacteria pathogenicity. Therefore, studies aimed at finding and characterizing bioactive compounds with antibiofilm activity is necessary, as an alternative step in overcoming this problem.

Natural products have become the source of novel therapeutics discovery, hence microbes as one of them have also become the primary source of drug discovery [9]. Actinomycetes are Gram-positive bacteria which are found in nature. These bacteria are known to produce many bioactive compounds [10]. Based on our previous studies, we found extracts of actinomycetes isolates which showed both inhibitory and destructive antibiofilm towards biofilm structure of Gram-positive and Gram-negative associated bacteria, such as *Pseudomonas aeruginosa*, *Salmonella typhimurium*, *Vibrio cholera*, *Bacillus cereus*, and *Staphylococcus aureus*. In addition, these extracts also produced quorum quenching compounds [11]. Therefore, in this study we screen the antibiofilm and anti-quorum sensing activity of these extracts on several aquaculture pathogenic bacteria.

## Result

### Bacterial cultivation

Sixteen isolates of Actinomycetes recovered from marine environments from our previous study were cultivated in yeast malt extract agar (YMEA) + 1% calcium carbonate. All of these isolates were attached on agar media and their colonies showed calcification due to the addition of calcium carbonate.

### Screening of anti-quorum sensing activity

**Fig. 1:**
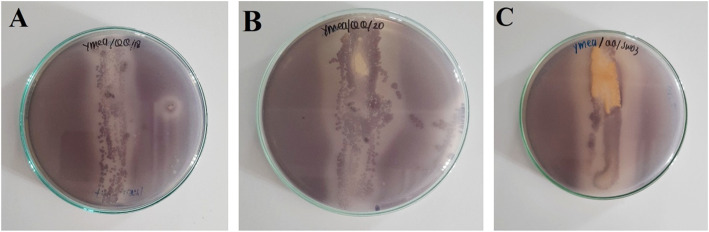
Result of screening of anti-quorum sensing activity of several Actinomycetes isolates (**a**: 18 PM; **b**: 20 PM; **c**: SW03)

All of the isolates showed anti-quorum sensing activity against indicator bacteria, namely *Chromobacterium violaceum* wild-type. It is characterized by the translucent zone around the straight streak area of the isolate (Fig. 1) (Table 1). Therefore, these isolate were used in the further assay.

**Table 1:** Result of detection of anti-quorum sensing activity and anti-bacterial activity assay of Actinomycetes isolates extracts (20 mg/mL)

Isolates	Detection of QS inhibitors	Antibacterial activity assay		
		AH	VH	SAG
TB12	**+++**	–	–	–
KP110	**++**	–	–	–
1 AC	**+++**	–	–	–
SW03	**+++**	–	–	–
CW17	**+**	–	–	–
16 PM	**++**	–	–	+ (D = 2.2 cm)
12 AC	**+++**	+ (D = 2 cm)	–	–
20 PM	**+++**	–	–	+ (D = 1.5 cm)
18 PM	**++**	–	–	+ (D = 1.2 cm)
14 PM	**+++**	–	–	+ (D = 2.1 cm)
CW01	+	–	–	+ (D = 2.5 cm)
SW17	**+**	–	+ (D = 2 cm)	–
SW16	**++**	–	–	–
SW14	**+++**	–	–	–
15 PM	**+++**	–	–	+ (D = 1.1 cm)
11 AC	**+**	–	–	–

*VH V. harveyi*, *AH A. hydrophila*, *SAG S. agalacatiae* ATCC 27956, *D* Diameter of clear zone

### Detection of anti-quorum sensing activity

**Fig. 2:**
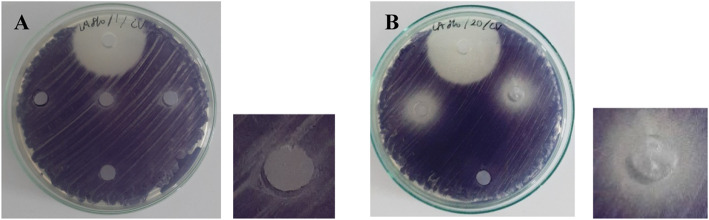
Result of detection of anti-quorum sensing activity of **a**) Isolate 1 AC **b**) Isolate 20 PM against *C. violaceum* wild-type.*The top well is a positive-control; well in the middle of the plate containing the extract of Actinomycetes isolates; and the lowest well is a negative-control

In this assay, all of the isolates which showed anti-quorum sensing activity in the previous assay were further tested. Extracts of Actinomycetes isolates (20 mg/mL) were spotted into each well. Table 1 showed that the result was varied. Based on the translucent zone around the well, the inhibitory activity was categorized into three: (+); (+)(+); (+)(+)(+) indicating weak; moderate; and strong inhibition, respectively (Fig. 2).

### Antibacterial activity assay

**Fig. 3:**
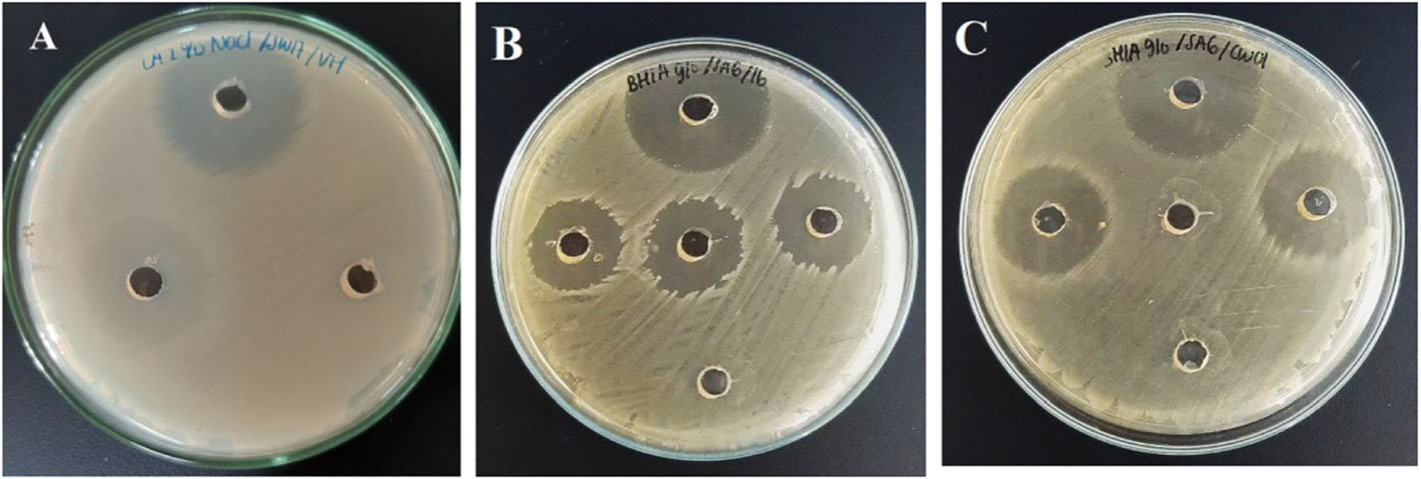
Result of antibacterial assay **a**) Isolate SW17 against *Vibrio harveyi* **b**) Isolate 16 PM against *Streptococcus agalactiae* **c**) Isolate CW01 against *Streptococcus agalactiae* .*The top well is a positive-control; well in the middle of the plate containing the extract of Aktinomycetes isolates; and the lowest well is a negative-control

Antibacterial assay was necessary to be performed to avoid false-positive results in antibiofilm activity assay (Fig. 3). In this assay, extracts of Actinomycetes isolates (20 mg/mL) were spotted into each well. Based on the result there were several isolates, which showed anti-bacterial activity, such as SW17 and 15, 14, CW01, 16, 18, and 20 against *V. harveyi* and *S. agalactiae*, respectively (Fig. 2) (Table 1). These isolates were ruled out in further assay.

### Antibiofilm activity assay

Antibiofilm activity assay was carried out to quantify the inhibitory and destructive activity of the isolates against pathogenic bacteria tested. Based on the result of the assay, most of the isolates showed higher inhibitory activity compared to their destructive activity. Table 2 showed that 16 PM, CW01, and CW17 showed highest inhibitory activity against each pathogenic bacteria tested: 85.11, 44.23, and 53.42% against *A. hydrophila, V. harveyi*, *S. agalactiae* respectively. While, CW17 and SW14 showed highest destructive activity against pathogenic bacteria tested: 74.47 and 51.88% against *A. hydrophila* and *V. harveyi*, respectively. In addition, based on this assay, all of the isolates work most effectively against specific pathogenic bacteria in specific mechanism of action (Fig. 3).

**Table 2:** Antibiofilm activity of extracts of Actinomycetes isolates (20 mg/mL) against aquatic pathogenic bacteria

Isolates	Inhibition (%)			Destruction (%)		
	VH	AH	SAG	VH	AH	SAG
TB12	28.77	78.67	26.34	36	22.62	18.09
KP110	26.37	34.49	27.90	0	15.22	42
1 AC	0	33.33	3.30	26.65	29.13	33.47
SW03	0	75.57	0	0	38.83	14.79
CW17	0	74.47	53.42	0	16.57	18.16
16 PM	36.66	85.11	–	0	17.31	–
12 AC	33.07	–	23.86	0	–	36.78
20 PM	0	82.44	–	0	28.46	–
18 PM	0	76.90	–	0	76.54	–
14 PM	32.94	78.60	–	0	14.54	–
CW01	44.23	76.10	–	23.52	59.75	–
SW17	–	37.99	27.23	–	25.02	27.99
SW16	24.08	27.45	0	0	3.83	0
SW14	35.29	12.62	0	51.88	21.35	0
15 PM	0	9.85	–	0	0	–
11 AC	35.99	79.17	71.52	39.87	23.54	36.33

*VH V. harveyi*, *AH A. hydrophila*, *SAG S. agalacatiae* ATCC 27956

### Determination of the bioactive compound

**Fig. 4:**
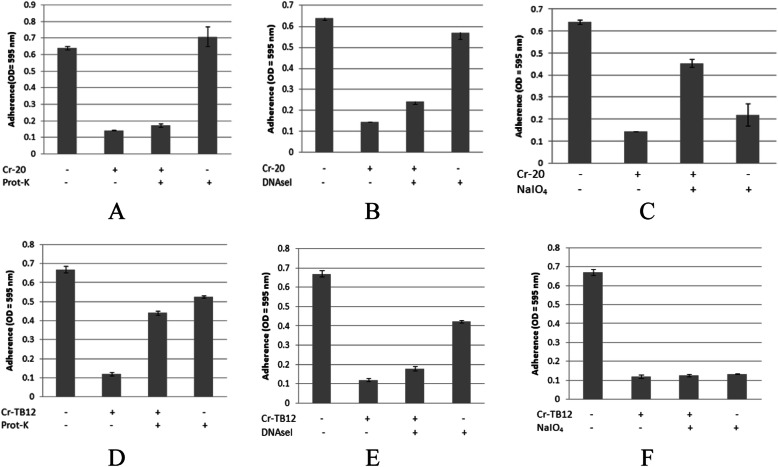
Characterization of extracts produced by isolates 20 PM (**a**, **b**, **c**) and TB12 (**d**, **e**, **f**) which is responsible for the inhibitory activity towards biofilm structure of *A. hydrophila* by treating the extract with NaIO_4_ (20 mM), proteinase K (1 mg/mL), and DNase I (100 mg/mL). *Cr = Crude extracts

**Fig. 5:**
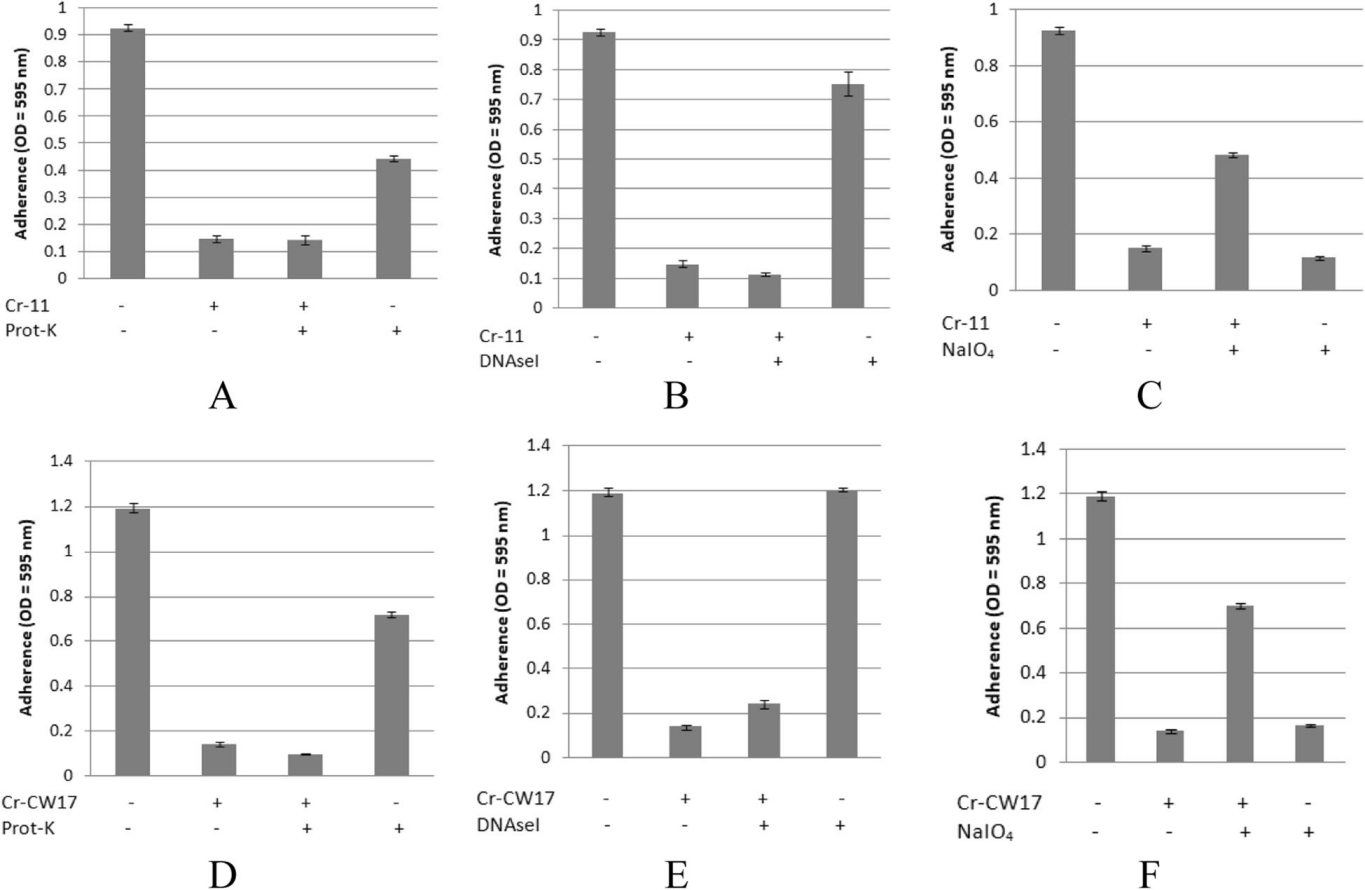
Characterization of extracts produced by isolates 11 AC (**a**, **b**, **c**) and TB12 (**d**, **e**, **f**) which is responsible for the inhibitory activity towards biofilm structure of *S. agalactiae* by treating the extract with NaIO_4_ (20 mM), proteinase K (1 mg/mL), and DNase I (100 mg/mL)

The determination of these compounds was performed by static inhibition assay using the crude extract of each Actinomycetes isolates. The pre-treatment using proteinase-K, nuclease, and NaIO_4_ could decrease the antibiofilm activity due to the destruction of active compounds, which is responsible to its antibiofilm activity. In this study, we found that polysaccharides are the most commonly found compounds which were responsible to their antibiofilm in extracts tested. Figure 4c showed that the pre-treatment of extract 20 PM using NaIO_4_ decrease its inhibitory activity towards biofilm structure of *A. hydrophila*. While pre-treatment using proteinase-K and nuclease showed relatively no effect to its antibiofilm activity (Fig. 4a and b). Similarly, Fig. 5c and f showed that pre-treatment of extracts 11 AC and CW17 using NaIO_4_ decrease their inhibitory activity towards biofilm structure of *S. agalactiae*. While pre-treatment using proteinase-K and nuclease showed no effect to its antibiofilm activity (Fig. 4a and b).

**Fig. 6:**
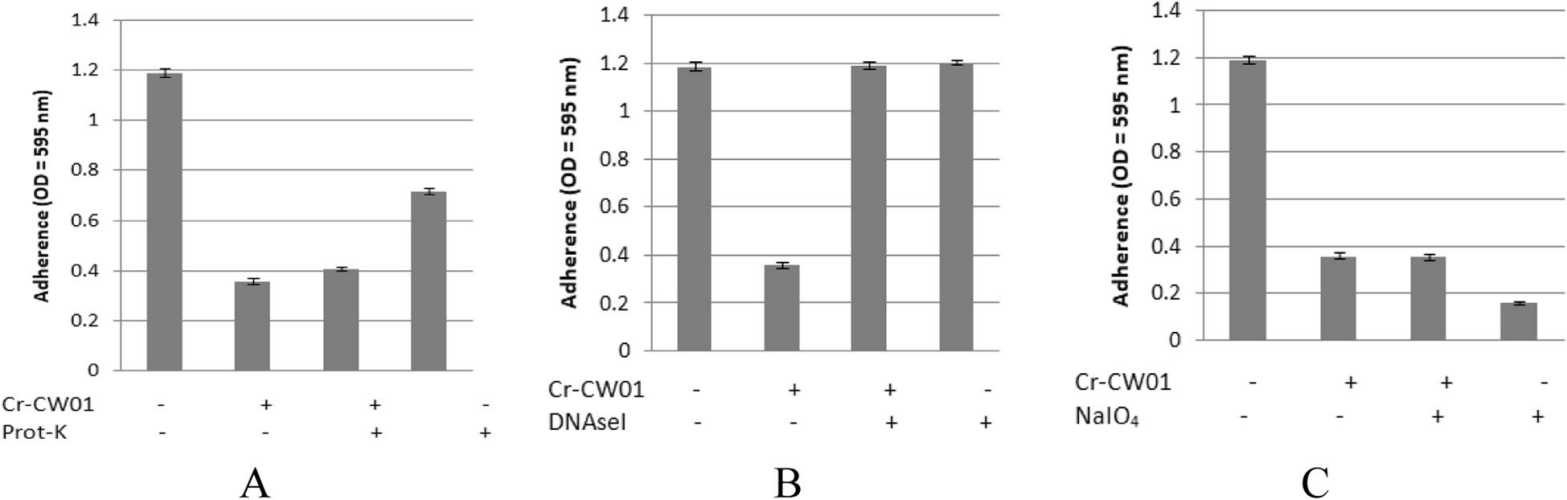
Characterization of extracts produced by isolates CW01 (**a**, **b**, **c**) which is responsible for the inhibitory activity towards biofilm structure of *S. agalactiae* by treating the extract with NaIO_4_ (20 mM), proteinase K (1 mg/mL), and DNase I (100 mg/mL)

In addition, Fig. 4d showed that pre-treatment using proteinase-K decrease inhibitory activity of extract TB12 towards biofilm structure of *A. hydrophila*. While, pre-treatment of nuclease and NaIO_4_ showed no effect to those activity (Fig. 4e and f), indicating that the compound which was responsible to this activity is protein. Other than that, we also found that nucleic acid might be another compound which was responsible to inhibitory activity of these extracts tested as seen in Fig. 6b, which showed that pre-treatment of CW01 using nuclease decrease those activity. While, pre-treatment using proteinase-K and NaIO_4_ showed no effect (Fig. 6a and c).

### Brine shrimp lethality assay

Seven potential Actinomycetes isolates extracts which showed the highest antibiofilm activity was tested using BSLA method. This assay was performed in five replicates and two different batches. Table 3 showed the LC_50_ value of these isolates, which were more than 1 mg/mL.

**Table 3:** The result of BSLA of several Actinomycetes isolates

Isolates	LC_50_ (mgμ/mL)
TB12	4.365
20 PM	8.511
CW17	2.979
CW01	3.917
11 AC	3.954
SW14	3.062
16 PM	2.773

## Discussion

Quorum sensing is an inter-cell communication system, which aims to regulate gene expressions from cell communities by producing, detecting, and responding to self-synthesized small signal molecules, called autoinducers [12]. Many previous studies reported the involvement of this system in the bacterial virulences, some of them are the formation, maintenance, and dispersal of biofilm structures. Therefore, one approach that is considered promising is to target this system with the aim of either inhibiting or destructing biofilm structures [13]. Based on the result of screening of anti-quorum sensing activity, we found sixteen Actinomycetes isolates which showed anti-quorum sensing activity. This is confirmed by the result of the detection of anti-quorum sensing activity, that the extract of these isolates showed inhibitory activity against violacein pigment production of reporter strain *C. violaceum* wild-type without affecting their growth. Given this information, these isolates were further tested against aquatic pathogenic to confirm whether their anti-quorum sensing activity could suppress the pathogenic bacteria tested.

In this study, we also found that several Actinomycetes isolates showed anti-bacterial activity, such as SW17 and CW01 against *V. harveyi* and *S. agalactiae*, respectively. In this case, these isolates were not used in further assay, due to the false-positive result which potentially could happen. In addition, antibiofilm non-biocidal strategies target bacterial behavior rather than bacterial fitness. Therefore, they are less likely causing resistance in bacteria [14].

The antibiofilm assay showed varied results. Most of the isolates showed promising antibiofilm activity against specific pathogenic bacteria in specific mechanism of action. For example, isolate 16 PM showed 85.11% inhibitory activity against *A. hydrophila*. Given the information from previous assay, that these isolates showed anti-quorum sensing activity then it is possible that these isolates might produce quorum sensing inhibitors (QSI) which interfere the quorum sensing system of pathogenic bacteria tested which resulting in inhibition and destruction of their biofilm structure. QSI might work in three manners, namely 1) inhibiting the production of signaling molecules; 2) directly degrading signal molecules; and 3) inhibiting the binding of signaling molecules to the receptors. In addition, QSIs are considered as specific antibiofilm molecules since each bacteria have different QS systems [13].

The nature of QSIs could be enzymatic or non-enzymatic. In the case of enzymatic, signal molecules might be enzymatically degraded by Acylhomoserine lactone (AHL) lactonase and AHL acylase, hence prevent their accumulation and subsequent activation of their QS systems. Several reports have reported that some bacteria, such as *Bacillus*, *Klebsiella pneumonia*, *Pseudomonas aeruginosa*, and *Streptomyces* sp. M664 showed ability to degrade AHL signal molecules of other bacteria by secreting enzymes, such as lactonase and acylases. These enzymes are known to be able to hydrolyze homoserine lactone (HSL) ring and amide bonds of AHL molecules, respectively [15]. In addition, based on the study conducted by [16], they found that *Shewanella* sp. MIB015 showed obvious degrading activity against AHL-mediated production of exoprotease in *Aeromonas* sp. Similarly, other study also reported that by mutating *ahyI*, a gene coding for AHL signal synthesis enzymes, resulted in the formation of unstructured biofilm in *Aeromonas hydrophila* [17]. All of these data suggest that by interfering the quorum system of these pathogenic bacteria, it might lead to interference of their virulence one of them is formation of biofilm structure.

In this study, we also performed characterization of bioactive compound by treating several selected extracts with protease, DNAseI, and NaIO_4_. Protease and nuclease break down protein and nucleic acid, respectively. While, NaIO_4_ is known to be able to hydrolyze polysaccharides by oxidizing the carbons bearing vicinal hydroxyl groups and cleaving the C-C bonds [18]. In this case, we expected that treatment of these extracts were going to digest the responsible compound thereby caused a decrease of antibiofilm activity. The result of this assay was varied. In this case, several extracts such as 20 PM, 11 AC, and CW17 showed a decrease of antibiofilm activity after being treated with NaIO_4_. Thereby, we could indicate that the responsible compounds in those extracts were polysaccharides. Other than that, we also found that treatment other extract with protease-K and nuclease resulted in a decrease of antibiofilm activity indicating that the responsible compounds of these extracts are protein and nucleic acid, respectively.

Polysaccharides have been reported to be one of the most common antibiofilm agents. Most of them act as surfactant altering the properties of abiotic and biotic surfaces. In this case, polysaccharides are known to be able to modify the wettability, charge, and hydrophobicity of the surface, hence interfere the interaction between cell-surface. Valle et al. (2006) [19] reported that the treatment of abiotic surfaces with group II capsular polysaccharides led to lowering the interfacial energy and hydrophobicity of the glass surfaces. This caused drastic reduction of initial adhesion and biofilm maturation of wide range of Gram-positive and Gram-negative bacteria. Similarly, Jiang et al. (2011) [18] also reported that exopolysaccharide isolated from marine bacterium *Vibrio* sp. showed inhibitory activity against cell aggregation of *P. aeruginosa*. In addition, this compound also able to disrupt pre-formed biofilm structure of *P. aeruginosa*. These data suggest that polysaccharides not only could weaken the cell-surface interactions (initial adhesion) but also could interfere cell-to-cell interactions (biofilm maturation). In addition, others modes of action of polysaccharides as antibiofilm agent have been reported, such as down-regulating genes which related to biofilm formation and inducing cellular motility [20].

Protein is also known to be one of antibiofilm agents. Biofilm dispersal is a process which involves destruction of EPS mediated by various matrix-degrading enzymes, such as proteases and deoxyribonuclease (DNAses). The degradation of EPS causes the maturation process on biofilm to be disrupted increasing the sensitivity of the sessile cells to antibiotics and host immune system [21]. Another antibiofilm agent which also found in this study was extracellular DNA (eDNA). This activity was also reported by study conducted by [22], which stated that eDNA secreted by *Caulobacter crescentus* showed an ability to act as anti-adhesive agent by coating the adhesive surface, thereby inhibiting the initial cell attachment which is required for biofilm formation. In this case, most of these antibiofilm agents work specifically towards particular pathogens in specific mechanism of action.

Brine shrimp lethality assay is a simple, high-throughput cyto-toxicity assay. This assay is based on the killing ability of the compound tested on organism- brine shrimp (*Artemia salina*). This assay is used as preliminary assay for further toxicity experiment on mammalian animal models. Because it is easy, inexpensive, rapid, give a repetitive result, and accommodates a large number of nauplii for statistical validation. In this assay, we used *A. salina* as an object for determination of toxicity of compounds tested [23].

In this assay, we determine the value LC_50_ of each compound tested. LC_50_ is defined as estimation of the exposure concentration causing 50% of mortality of *A. salina* in specified period of time. If the LC_50_ value of compound tested is more than 1 mg/mL, then the compound is considered non-toxic. Conversely, if the LC_50_ value of compound tested is less than 1 mg/mL, then the compound is considered toxic [24]. In this study, we found that the LC_50_ value of all of the extracts tested were more than 1 mg/mL. Thereby, all of the extracts tested did not have cyto-toxic effect against *A. salina* nauplia. However, these extract needs to be further tested using more advance toxicity assay to ensure their toxicity property.

## Conclusion

In this study, all of the extracts of Actinomycetes isolates, such as 20 PM, CW01, and 11 AC showed potential inhibitory activity towards biofilm structure of all pathogenic bacteria tested. In addition, these extracts also showed anti-quorum sensing activity against reporter strain *C. violaceum* wild-type. We also found through characterization assay that most of the compound contained in these extracts which responsible for their antibiofilm activity were polysaccharides. We also found through characterization assay that most of the compound contained in these extracts which responsible for their antibiofilm activity were polysaccharides. Therefore, we concluded that these extracts are considered to be promising to combat biofilm-related-infections in aquaculture industry. However, further research about optimization of extract concentration to exhibit better destructive activity towards biofilm structure of all pathogenic tested and more advance toxicity assay are considered necessary to be performed.

## Methods

### Bacterial cultivation

In this study, sixteen Actinomycetes isolates recovered from marine environment, from our previous study and *Chromobacterium violaceum* WT wild-type were obtained from Atma Jaya Culture Collection. Whereas, *Vibrio harveyi* and *Aeromonas hydrophila* were obtained from health aquatic organism laboratory of Department of Aquaculture, Faculty of Fisheries and Marine Sciences, Bogor Agricultural Univeristy. *Streptococcus agalactiae* which were used in this study had been characterized as ATCC 27956.

Cryopreservation isolates of Actinomycetes were streaked onto Tryptic Soya Agar (TSA) (Oxoid) and incubated at 28 °C for 7 days. Afterwards, single colony was picked, streaked onto yeast malt extract agar (10 g Malt extract; 4 g Yeast extract; 4 g Glucose; 2 g CaCO_3_; 1 L distilled water) (YMEA), and incubated at 28 °C for 7 days. Whereas, pathogenic bacteria were streaked onto luria agar (5 g NaCl; 5 g Yeast extract; 10 g Tripton; 20 g Agar Bacto; 1 L distilled water) (LA) and incubated at 28 °C for overnight.

### Primary screening of anti-quorum sensing activity

This primary screening was performed based on Abudoleh and Mahasneh (2017) [25] method with some modifications. Firstly, Actinomycetes isolates were straight-streak onto LA and incubated at 28 °C for 3 days. Subsequently, *C. violaceum* WT wild-type as reporter strain was grown in luria broth (LB) and incubated at 28 °C for overnight. This culture was diluted in sterile LB until the absorbance value reaches 0.132 at 600 nm (McFarland 0.5). Then, 100 μL of the culture was put into 3 mL semisolid LA (0.75% w/v agarbacto). After incubation time of Actinomycetes isolates, the culture was poured onto the LA as an overlay. These plates were incubated at 28 °C for another 4 days. The result was observed. In this case, positive result was indicated by the inhibition of violacein pigment around the Actinomycetes isolates. Conversely, negative result was indicated by the production of violacein pigment around Actinomycetes isolates.

### Fermentation conditions and extracts preparation

Fermentation and extraction was performed using Balasubramanian et al. (2017) [26] with some modifications. Isolates of Actinomycetes were inoculated into Tryptic soy broth (TSB) + 1% glucose and incubated at 28 °C for 7 days at 150 rpm using rotary shaker. Afterwards, the culture was centrigufed at 6900 x g for 15 min. Supernatant was harvested and an equal volume of ethyl acetate was added. The supernatant was incubated at 28 °C for overnight at 150 rpm using rotary shaker. Then, solvent layer was harvested and evaporated using rotary evaporator to generate extracts. These extracts were dissolved in 1% (v/v) dimethyl sulfoxide (DMSO) to generate final concentration (20 mg/mL). The extracts were used for further assays.

### Detection of anti-quorum sensing activity

This step was performed based on the method published by Rajivgandhi et al. (2018) [27] with some modifications. *Chromobacterium violaceum* wild-type was streaked continuously in three directions onto LA using sterile cotton buds. Then, sterile cork borer was used to remove plugs of the agar. This plug was replaced by extract of Actinomycetes isolates. In this assay, DMSO was used as control negative, while Streptomycin (10 mg/mL) was used as positive control. Plates were incubated at 28 °C for overnight. Finally, translucent zone against violacein pigmentation of *C. violaceum* wild-type was observed.

### Anti-bacterial assay

Antibacterial assay was performed by using diffusion method according to Bauer et al. (1966) [28] with some modifications. Pathogenic bacteria were inoculated into LB and incubated at 28 °C for overnight at 150 rpm using rotary shaker. Afterwards, these cultures were diluted into sterile LB until the absorbance value reaches 0.132 at 600 nm (McFarland 0.5).

Pathogenic bacteria were streaked in three directions onto Mueller Hinton Agar (MHA) using sterile cotton buds. Afterwards, a sterile cork borer was used to remove the agar plugs. Then, these plugs were replaced by the extract of Actinomycetes isolates. In this assay, DMSO was used as control negative, while Streptomycin (10 mg/mL) was used as positive control. Plates were incubated at 28 °C for overnight. Then, clearing zone was observed. The isolate which showed anti-bacterial activity was ruled out in antibiofilm activity assay.

### Antibiofilm assay

Antibiofilm assay was performed in two categories based on their mechanisms, namely inhibitory and destructive activity assay. Preparation of this assay was performed, by inoculating pathogenic bacteria into LB medium. Then, cultures were incubated at 28 °C for overnight and diluted into sterile LB until the absorbance values reaches 0.132 at 600 nm (McFarland 0.5).

Antibiofilm assay was performed according to O’Toole and Kolter (1998) [29] with some modifications. For inhibitory activity assay, 200 μL of pathogenic bacteria suspension and Actinomycetes isolates extracts was loaded simultaneously into 96-well polystyrene plates. Then, plates were incubated at 28 °C for overnight, then staining process was performed. Conversely for destructive activity assay, firstly 200 μL of pathogenic bacteria suspension was loaded into 96-well polystyrene plates. Plates were incubated at 28 °C for overnight and Actinomycetes isolates extracts were loaded, then staining process was performed. For both assay, pathogenic bacteria suspensions were used as positive control, while sterile LB was used as negative control.

The process of staining was performed un-aseptically. Planktonic cells and media were discarded. Then, adherent biofilm cells were rinsed with water twice and air-dried for 30 min. These cells were stained by using 200 μL of 0.4% (b/v) crystal violet for 30 min. Then, crystal violet was discarded. These stained cells were rinsed with water for five times to remove any remaining crystal violet and air-dried for 30 min. Then, 100 μL of absolute ethanol was added and re-suspended to dissolve the stained cells. Subsequently, this ethanol was transferred into new 96-well polystyrene plates. The absorbance of each well was measured with microplate reader. Finally, the percentage of antibiofilm activity of Actinomycetes isolates extract was calculated using the formula below [30]:

$$ \%\mathrm{Activity}=\frac{\mathrm{OD}\ \mathrm{Positive}\ \mathrm{control}-\mathrm{OD}\ \mathrm{sample}}{\mathrm{OD}\ \mathrm{positive}\ \mathrm{control}}\ \mathrm{x}\ \left(100\%\right) $$

### Determination of the bioactive compounds

This method was based on this research (Jiang et al. 2011) [18] with some modifications. Some selected extracts of Actinomycetes isolates were treated with proteinase K (1 mg/mL), DNase I (100 μg/mL), and NaIO_4_ 20 mM at 37 °C for 12 h. Then, the post-treated extracts were used for biofilm inhibition and destruction activity assay. Finally, the activity of pre and post-treated extracts were measured and compared.

### Sample preparation for brine shrimp lethality assay (BSLA)

Samples were prepared by dissolving 1 mL extract of Actinomycetes isolates in 100 mL artificial sea water in order to get a stock solution (1000 ppm). Then, stock solution was diluted into final concentration (10, 100, 500, and 1000 ppm).

### Hatching the shrimp

This method was based on this research (Krishnaraju et al. 2006) [31] with some modifications. Three mg of brine shrimp eggs was added into $\frac{3}{4}$ volume of 1 L mineral water bottle filled with artificial sea water. Moreover to optimize the hatching process, the growing media was constantly aerated with air pump and illuminated with lamp. After 24 h, the nauplia were collected by pipette and put into tubes.

### Bioassay

Bioassay was performed according to the method published by Ramachandran et al. (2011) [32] with some modifications. Ten nauplia were transferred into tubes filled with 4.5 mL artificial sea water. Then, 500 μL of extracts was added into each tube. Tubes were maintained under illumination for 24 h at room temperature. Finally, the death percentage of nauplii was determined using this formula below:

$$ \%\mathrm{Death}=\frac{\mathrm{Death}\ \mathrm{nauplii}}{\mathrm{Total}\ \mathrm{nauplii}}\ \mathrm{x}\ \left(100\%\right) $$

In case the negative control does not give 0% of mortality, the formula was corrected using Abotts’s formula below:

$$ \%\mathrm{Death}=\frac{\mathrm{Death}\ \mathrm{nauplii}\ \mathrm{in}\ \mathrm{tested}\ \mathrm{vial}-\mathrm{death}\ \mathrm{nauplii}\ \mathrm{in}\ \mathrm{control}\ \mathrm{vial}}{\mathrm{Death}\ \mathrm{nauplii}\ \mathrm{in}\ \mathrm{control}\ \mathrm{vial}}\mathrm{x}\ \left(100\%\right) $$

In this assay, 500 μL of K_2_Cr_2_O_7_ was be used as positive control, while 500 μL of artificial water was used as negative control. The lethal concentration (LC_50_) was collected.

## Data Availability

All data generated or analysed during this study are included in this published article.

## Abbreviations

EPS: Extracellular polymeric substances
YMEA: Yeast Malt Extract Agar
QSI: Quorum Sensing Inhibitor
AHL: Acylhomoserine lactone
HSL: Homoserine lactone
LA: Luria Agar
LB: Luria Broth
TSB: Tryptic Soy Broth
DMSO: Dimethyl Sulfoxide
MHA: Mueller Hinton Agar
QS: Quorum Sensing
BSLA: Brine Shrimp Lethality Assay
